# Structural Basis for Tetramerization of *Klebsiella pneumoniae* *N*-Acetylglucosamine-6-Phosphate Deacetylase

**DOI:** 10.4014/jmb.2505.05019

**Published:** 2025-08-26

**Authors:** So Yeon Lee, Hyun Ho Park

**Affiliations:** 1 College of Pharmacy, Chung-Ang University, Seoul 06974, Republic of Korea; 2 Department of Global Innovative Drugs, Graduate School of Chung-Ang University, Seoul 06974, Republic of Korea

**Keywords:** Crystal structure, NagA, amidohydrolase superfamily, Klebsiella pneumoniae

## Abstract

N-acetylglucosamine-6-phosphate deacetylase (NagA) is a conserved enzyme involved in bacterial amino sugar metabolism, catalyzing the conversion of GlcNAc-6-phosphate to GlcN-6-phosphate and acetate. While NagA typically function as dimers, its quaternary diversity across species remains underexplored. Here, we present the crystal structure of *Klebsiella pneumoniae* (kpNagA), which forms a homotetrameric assembly both in crystal and in solution, as confirmed by SEC-MALS. Each monomer adopts a canonical (β/α)_8_ TIM barrel fold with a β-sandwich subdomain, and its active site, located around β10–β11 and α3–α4, coordinates a divalent zinc ion. Comparative analyses revealed conserved dimer interfaces but divergent tetrameric arrangements. Notably, *Pasteurella multocida* NagA also forms a stable tetramer, albeit via a distinct interface. These findings suggest species-specific tetramerization and broaden our understanding of NagA structural diversity and potential antibiotic targets.

## Introduction

*N*-acetylglucosamine-6-phosphate deacetylase (NagA) is a well-known member of the amidohydrolase superfamily (AHS) and serves as a key enzyme in bacterial amino sugar metabolism [1, 2]. NagA catalyzes the conversion of *N*-acetylglucosamine-6-phosphate (GlcNAc-6P) to glucosamine-6-phosphate (GlcN-6P) and acetate in a metal-dependent manner[3]. This reaction is essential for peptidoglycan recycling and the cell wall biosynthesis, making NagA indispensable for bacterial growth and survival [4-6]. NagA is highly conserved among bacteria, including numerous pathogenic species, and typically adopts (β/α)_8_ TIM (triose-phosphate isomerase) barrel fold with an additional β-barrel domain and a metal center critical required for catalytic activity [1, 6-8].

Despite their overall conservation, NagA enzymes exhibit notable diversity in their oligomeric assemblies [7-10]. Most bacterial NagA homologs exist predominantly as dimers in solution, which is recognized as the minimal assembly required for catalytic activity, as demonstrated by biochemical and structural analyses [11, 12]. Although tetrameric assemblies are sometimes observed in crystal structures, these often result from symmetry-related packing or weak interfaces and do not necessarily represent the physiological state [7, 13]. In many cases, crystallographic tetramers are not supported by solution studies and are considered artifacts.

An exception is NagA from *Pasteurella multocida*, which forms a stable tetramer both in crystal and in solution, though the quaternary interfaces differ among species [14]. *Escherichia coli* NagA has been reported in two different crystal forms: in one (PDBID: 2P50), the solution oligomeric state remains undetermined, while in another (PDBID: 1YRR), small-angle-X-ray scattering (SAXS) confirmed a tetramer in solution, even though only a dimer is present in the asymmetric unit [7, 12].

In this study, we reveal the tetrameric structure of NagA from *Klebsiella pneumoniae*, a clinically important multidrug-resistant pathogen [15]. Since NagA is essential for cell wall biosynthesis, our results structural insights relevant to antibiotic targeting in superbugs like *K. pneumoniae* [16, 17]. Comparative analysis demonstrates that, while kpNagA and pmNagA both display stable tetramers, they exhibit distinct dimer-dimer interfaces. Analysis of the kpNagA interface reveals features not seen in other bacterial NagAs, highlighting the structural diversity and evolutionary adaptation within enzyme family.

## Material and Methods

### Protein Expression and Purification

The gene encoding *K. pneumoniae* NagA (accession code: WP_274787561.1) was synthesized (Bionics, Republic of Korea) and inserted into the pET28a vector (Novagen, USA) using NdeI and XhoI restriction sites. The resulting plasmid included N- and C-terminal 6XHis tags to aid purification. Recombinant construct was transformed into *E. coli* BL21(DE3) cells. A single colony was grown overnight in LB medium containing 50 μg/ml Kanamycin at 37°C. The culture was then diluted 1:200 into fresh LB with antibiotic and incubated at 37°C until OD_600_ reached 0.7–0.8. Expression was induced with 0.25 mM IPTG, followed by incubation at 20°C for 18 h with shaking.

Cells were harvested by centrifugation (3,500 g, 15 min, 4°C) and resuspended in lysis buffer (20 mM Tris-HCl, pH 8.0, 500 mM NaCl, 25 mM imidazole). Lysis was performed by sonication on ice, and the lysate was clarified by centrifugation (14,000 g, 30 min, 4°C). The supernatant was incubated with Ni-NTA resin (Qiagen) for 2 h at 4°C, then loaded onto a gravity column and washed with buffer containing 60 mM imidazole. Proteins were eluted using 250 mM imidazole in the same buffer. Eluted fractions were pooled and further purified by size-exclusion chromatography (Superdex 200 Increase 10/300 GL, GE Healthcare, USA) equilibrated in 20 mM Tris-HCl, pH 8.0, 150 mM NaCl. Final protein concentration was adjusted to 6.1 mg/ml. Purity was assessed by SDS-PAGE.

### Crystallization and Data Collection

Crystallization was performed by hanging-drop vapor diffusion at 20°C. Equal volumes of protein solution (6.1 mg/ml) and reservoir (initial: 20% PEG 3350, 0.2 M potassium formate; optimized: 22% PEG 3350, 0.25 M potassium formate, 0.01 M Strontium chloride hexahydrate) were mixed and equilibrated against reservoir solution. Crystals were cryoprotected in reservoir supplemented with 30% glycerol. X-ray data were collected at -178°C on beamline BL-5C at Pohang Accelerator Laboratory (Republic of Korea). Data were processed with HKL2000 [18].

### Structure Determination and Analysis

The structure was solved by molecular replacement using PHASER (PHENIX suite) [19], with AlphaFold2-generated [20] NagA models as search templates. Model building and refinement were performed using AutoBuild (PHENIX) [21], COOT [22], and phenix.refine [23], with quality validated in MolProbity [24]. All structural images were prepared in PyMOL [25].

### SEC-Multi-Angle Light Scattering (MALS) Analysis

Absolute molecular mass of kpNagA in solution was determined by size-exclusion chromatography coupled to multi-angle light scattering (SEC-MALS) [26]. Purified protein was run on a Superdex 200 Increase 10/300 GL column (24 ml, GE Healthcare) at 0.5 ml/min at 20°C, using a DAWN-Treos detector (Wyatt Technology, USA) connected to an ÄKTA explorer system (GE Healthcare). BSA was used for calibration. Data analysis was performed with ASTRA software (Wyatt Technology).

### Sequence Alignment

Multiple sequence alignment of NagA proteins was performed using Clustal Omega [27].

## Results and Discussion

### Structural Characterization of monomeric *Klebsiella pneumoniae* NagA

Recombinant kpNagA was efficiently expressed in *E. coli* and purified to homogeneity by Ni-NTA affinity and size-exclusion chromatography. SEC analysis revealed two major elution peaks, 1^st^ peak eluted around 10 ml, and the 2^nd^ peak eluted around 12~13 ml (Fig. S1A). SDS-PAGE analysis showed a single clear band at ~42 kDa, corresponding to the expected monomeric mass, both at 1^st^ peak and 2^nd^ peak (Fig. S1B). The thickness of the band width which show the amount of protein, and the purity were better at 2^nd^ peak elution, so 2^nd^ peak was determined as the major peak while 1^st^ peak determined as the higher oligomeric state peak of NagA. SEC analysis revealed a major elution peak (2^nd^ peak) with an apparent molecular mass consistent with a homotetramer, as confirmed by calibration with molecular weight standards (Fig. S1C).

**Fig. 1:**
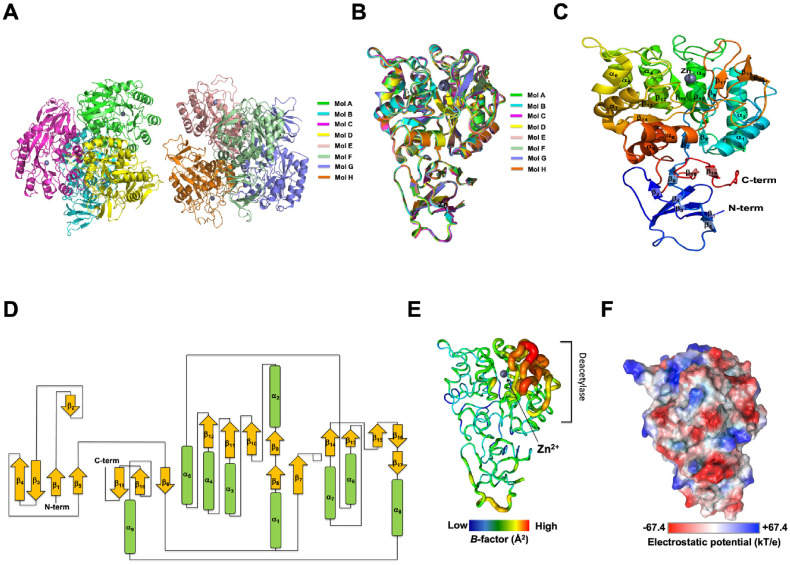
Crystal structure of NagA derived from *Klebsiella pneumoniae*. (**A**) Cartoon representation of crystal structure kpNagA reveals eight molecules in the asymmetric unit, forming two tetramers. Each monomer is shown in a different color. (**B**) Superimposition of the eight monomers illustrates high structural conservation among chains. (**C**) Structure of monomeric subunit, colored from blue (N-terminus) to red (C-terminus) with secondary structures annotated. (**D**) Topology diagram of kpNagA indicating the connectivity of α-helices, β-strands and loops. (**E**) *B*-factor putty representation of kpNagA. Warmer colors indicate regions of higher atomic displacement. The most flexible region, corresponding to the deacetylase domain, is indicated. (**F**) Electrostatic surface potential map of kpNagA, colored from negative potential (red, -67.4kT/e) to positive potential (blue, +67.4kT/e).

The crystal structure of kpNagA was determined, revealing two homotetrameric assemblies with Zn ions bound to each monomer (Fig. 1A). Detailed diffraction data and refinement statistics are summarized in Table 1. Structural comparison among the eight molecules presents in the asymmetric unit demonstrated high conservation, with RMSD of 0.55Å (Fig. 1B). The zinc-binding site consistently preserved across all molecules. Each kpNagA monomer is composed of 9 α helices and 19 β strands, adopting the canonical (β/α)_8_ TIM barrel fold characteristic of NagA enzyme (Fig. 1C). Eight parallel β strands (β7-β14) alternate with eight α helices (α1-α8), forming a central β sheet core encased by surrounding helices (Fig. 1C). This barrel architecture is a hallmark of the amidohydrolase superfamily and is essential for maintaining the structural integrity of the active site [1]. Following the eight β strands and seven α helices, an insertion of three additional β strands (β15-β17) occurs, followed by the final eighth α helix, completing the TIM barrel structure (Fig. 1C and 1D). The Zn ion is coordinated by residues from two α-helices (α3 and α4) and two β-strands (β10 and β11), located at the center of the TIM barrel, comprising the catalytic core of the deacetylase active site (Fig. 1C).

Beyond the TIM barrel, additional structure features contribute to the overall fold of NagA. The N-terminal region contains six β-strands (β1–β6) that assemble into an antiparallel β-sheet, while the C-terminal region contributes two β-strands (β18–β19) (Fig. 1C and 1D). Together, these β-strands form a β-sandwich fold that flanks the TIM barrel domain and likely stabilizes the overall architecture.

The average B-factor of the monomeric NagA structure was 87.46Å^2^, with increased flexibility observed in the region surrounding the insertion of three additional β strands (β15-β17) near the active site (Fig. 1E). This localized flexibility may facilitate conformational changes associated with the active state and could correspond to the putative substrate-binding region. Electrostatic surface potential analysis, ranging from ±67.4kT/e, revealed an even distribution of positive and negative charges across the molecular surface (Fig. 1F).

### kpNagA Exhibits Tetrameric Formation in Solution

Previous genetic and biochemical studies have indicated that a dimeric assembly represents the minimal functional unit for catalytic activity within the NagA family [9-12]. However, whether this dimeric form is universally conserved across all NagA homologs, including that of *K. pneumoniae* (kpNagA), has not been clearly established [16].

**Fig. 2:**
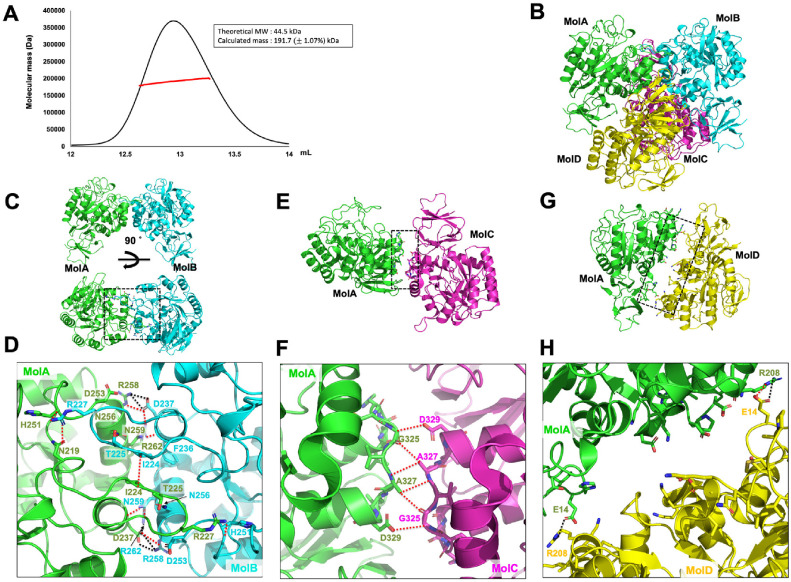
Dimeric structure of kpNagA and its interface analysis and conformation. (**A**) SEC-MALS profile confirms kpNagA forms a tetramer in solution, with a calculated molecular mass of 191.7 kDa. (**B**) Overall quaternary structure of kpNagA showing a dimer-of-dimers arrangements. (**C**) Symmetry view of the MolA-MolB interface with two orientations. (**D**) Magnified view of the MolA-MolB interface showing extensive hydrogen bonding (red dashed lines) and salt bridges (black dashed lines). (**E**) Symmetry view of the MolA-MolC interface. Protein-protein interactions (PPIs) within the dimer are marked with black dashed boxes. (**F**) Magnified view of the MolA-MolC interface, highlighting hydrogen bonds (red dashed lines). (**G**) MolA-MolD dimer viewed along the interface plane. (**H**) Magnified view of the MolA-MolD interface, highlighting salt bridge formation (black dashed lines).

To determine the oligomeric state of kpNagA in solution, we employed SEC coupled with multi angle light scattering (SEC-MALS). The major elution peak corresponded to an absolute molecular mass of 191.7 kDa, with a fitting error of 1.07% (Fig. 2A). Given that the theoretical molecular mass of a monomeric kpNagA with terminal His-tags is 44.5 kDa, these data strongly support that kpNagA predominantly forms a tetramer in solution.

Examination of the crystallographic asymmetric unit revealed two tetrameric assemblies, each organized as a dimer of dimers (Fig. 2B). Protein-protein interfaces were further analyzed using the PDBePISA server. Among the detected interfaces, the dimer formed between Molecule A (MolA) and Molecule B (MolB) exhibited a PISA score of 1.000, indicating a highly probable biologically relevant interaction (Table 2 and Fig. 2C). This MolA-MolB interface buries 1019.4Å^2^ of surface area, corresponding to 6.76% of the total molecular surface, and involves 26 residues, approximately 6.5% of the total amino acids.

The MolA-MolB dimer, consistent with canonical NagA family features, is stabilized primarily by interactions between the α6 and α7 helices and their connecting loops [9-12]. A network of 23 hydrogen bonds and 9 salt bridges was observed at this interface, mediated by residues such as I224, D237, H251, R258, and R262 (Fig. 2C and 2D).

Additional contacts between MolA and MolC, and between MolA and MolD, were also identified. Although their PISA scores were low (0.000), suggesting weak individual interfaces, the SEC-MALS data and SEC analysis confirming a tetramer in solution implies that these interactions likely contribute to the overall stabilization of the tetrameric assembly (Figs. 2A and S1). The MolA-MolC interaction is mainly mediated by the connection loops between α8 and α9, forming several hydrogen bonds (Fig. 2E and 2F). The MolA-MolD interface includes electrostatic contacts, notably between E14 and R208 at a distance of approximately 3.5Å, forming a salt bridge (Fig. 2G and 2H).

The discrepancy between the low PISA scores and the stable tetramer observed in solution may, in part, reflect the limited resolution (3.67Å) of the crystal structure, which can hinder precise modeling of side chain interactions critical for interface assessment.

Together, these findings establish that kpNagA adopts a stable tetrameric architecture both in solution and in the crystal state. The MolA-MolB interface forms the core dimeric unit essential for structural and catalytic integrity, consistent with the conserved features of the NagA family. In addition, the secondary interfaces between MolA-MolC and MolA-MolD, also likely contribute to the overall stabilization of the tetrameric assembly. These structural insights highlight the plasticity of NagA oligomerization modes and provide a framework for understanding the functional significance of higher-order assemblies in amino sugar metabolism.

### Sequence Comparison of Various NagA Homologs

**Fig. 3:**
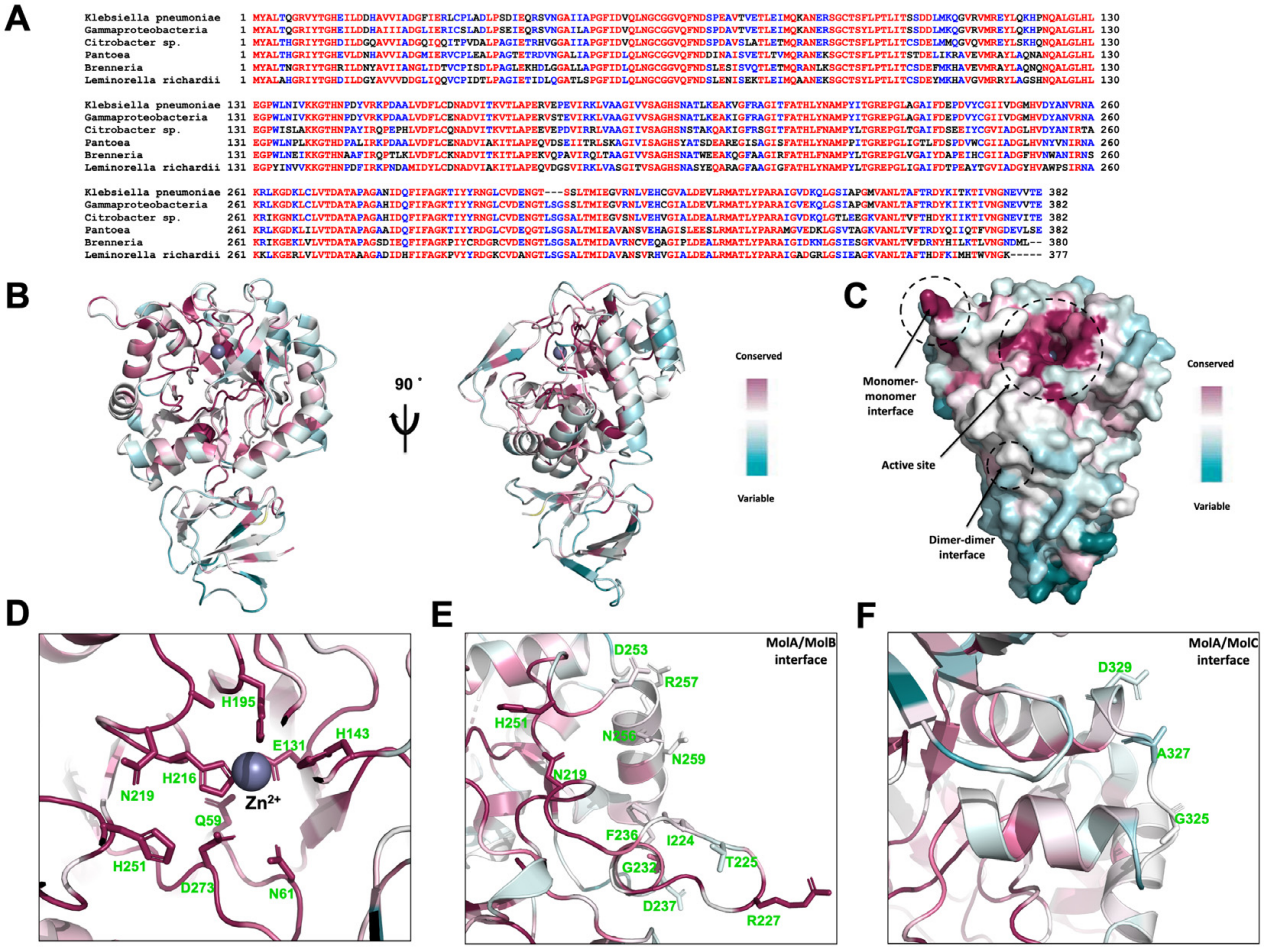
Sequence-based comparison of kpNagA homologs. (**A**) Multiple sequence alignment of kpNagA and its homologs using Clustal Omega. Fully and partially conserved residues are colored red and blue, respectively. (**B**) Conservation mapping of the kpNagA monomer calculated using the ConSurf server, displayed in cartoon representation. (**C**) Surface representation of kpNagA. The active site, monomer-monomer interface, and dimer-dimer interface are indicated with black dashed circles. (**D**) Close-up view of the active site. Conserved residues involved in Zn coordination and substrate binding are shown as sticks and labeled. (**E**) Magnified view of monomer-monomer interface. Key conserved residues contributing to dimerization are annotated. (**F**) Magnified view of dimer-dimer interface, between MolA-MolC.

To investigate the evolutionary conservation of kpNagA, multiple sequence alignment was performed using representative NagA homologs from diverse bacterial species. Most bacterial NagAs range from 370 to 390 amino acids in length (Fig. 3A). Residues forming the metal-binding and substrate-binding regions within the catalytic site were found to be strictly conserved across species (Fig. 3B and 3C). ConSurf analysis further confirmed that the active site region, including metal coordinating residues E131, H143, H195, H216 and substrate recognition residues N219, H251, and D273, is the most highly conserved surface region in kpNagA (Fig. 3C and 3D). These residues are predominantly located on β10–β11 and α3–α4, and their connecting loops, which together form the structurally conserved catalytic core of the NagA enzyme family [28]. In contrast, comparative analysis of residues involved in oligomerization revealed a distinct pattern. The interface residues forming the canonical MolA-MolB dimer such as N219, R227 and N219, were moderately to highly conserved, consistent with the established role of the dimer as the minimal functional unit among bacterial NagAs (Fig. 3C and 3E). However, residues contributing to dimer-dimer interactions in the MolA-MolC and MolA-MolD interfaces were poorly conserved, suggesting that the tetrameric assembly observed in kpNagA may represent a species-specific adaptation rather than a universally conserved feature (Fig. 3C and 3F).

These results support the view that dimerization is a conserved and functionally essential property of NagA enzymes, whereas tetramer formation may occur selectively in certain bacteria, possibly for structural stabilization or context-dependent regulation.

### Structural Comparison of Various NagA Homologs

**Fig. 4:**
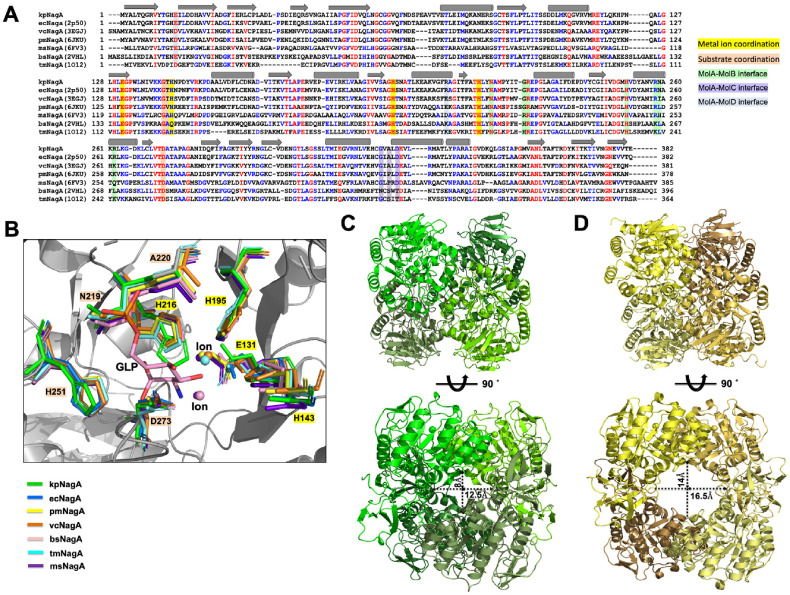
Structure-based comparison of kpNagA homologs. (**A**) Structure-based sequence alignment of kpNagA and homologs identified via the Dali server. Secondary structure elements and conserved functional residues are annotated: metal ion coordination (yellow), substrate coordination (orange), MolA-MolB interface (green), MolA-MolC (purple), and MolAMolD (blue). Each NagA homolog is labeled with a two-letter species code: *ec*, *Escherichia coli*; *vc*, *Vibrio cholerae*; *pm*, *Pasteurella multocida*; *ms*, *Mycobacterium smegmeatis*; *bs*, *Bacilus subtilis*; *tm*, *Thermotoga maritima*. (**B**) Magnified view of conserved catalytic site. Coordinating residues are shown as sticks, with ions and substrate molecules positioned based on bound structures. (**C**) Tetrameric structure of kpNagA showing a compact arrangement with a smaller central pore. (**D**) Tetrameric structure of pmNagA forming larger central cavity.

To investigate structural conservation and divergence within the NagA family, kpNagA was compared to structurally characterized homologs from multiple bacterial species using the DALI server. These homologs span a range of 27~86% sequence identity and share a common fold (Fig. 4A and Table 3). Structural superposition of monomeric subunits confirmed conservation of the overall architecture, including the (β/α)_8_ TIM barrel and associated β-sheet domains (Fig. S2A). Active site residues involved in metal coordination and substrate binding were strictly conserved across homologs, and the location of the bound ions was consistently maintained in all aligned structures (Fig. 4B) [7, 10, 28]. Structural alignment of dimeric assemblies revealed that kpNagA shares a conserved MolA-MolB dimer interface with *E. coli*, *P. multocida*, *T. maritima*, and *M. smegmatis* NagAs (Fig. S2B and S2C) [7, 13, 14]. In contrast, *V. cholerae* and *B. subtilis* NagA exhibited distinct dimer arrangements (Fig. S2B)[9, 29, 30]. Notably, the vcNagA dimer resembled the MolA-MolC interface of kpNagA, sharing conserved residues such as G233, A235, and D237 (Fig. S2D). Similarly, bsNagA displayed a unique dimer interface topologically similar to the MolA-MolD interface of kpNagA, although structural alignment was less precise (Fig. S2E). These observations suggest that alternative dimeric contacts observed in homologous species could support the additional inter-subunit interactions that stabilize the tetrameric architecture of kpNagA. While most NagAs are known to function as dimers in solution, ecNagA has been reported to form tetramers in solution based on SAXS data; however, its crystallographic tetramer’s detailed interface analysis remains limited [12]. Among the analyzed homologs, only pmNagA forms a stable tetramer both in solution and in the crystal structure, similar to kpNagA [14]. Despite this similarity, their tetrameric organizations are distinct: kpNagA forms a compact tetramer with all four subunits interacting, generating a smaller central cavity with a radius of approximately 8-12.5Å (Fig. 4C). In contrast, pmNagA assembles into a more open, ring-shaped tetramer, where each subunit interacts only with two adjacent partners, resulting in a wider central cavity with a radius of 14~16.5Å (Fig. 4D) [14].

Taken together, these findings reinforce the idea that dimerization is conserved and functionally essential feature across bacterial NagAs, forming the minimal active unit. In contrast, tetramerization appears to be species-specific, potentially serving auxiliary roles such as structural stabilization or regulatory control. Interestingly, higher-order oligomerization has not been observed in the human orthologs of NagA (PDBID: 7NUT), which are known to function as dimers, suggesting that evolution may have favored a streamlined assembly in higher organisms to optimize catalytic efficiency [10]. The structural diversity observed in tetrameric interfaces, such as those in kpNagA and pmNagA, therefore highlights the evolutionary plasticity of NagA quaternary architecture and may reflect adaptations to distinct cellular environments or regulatory demands.

Moreover, the structural characterization of kpNagA from a clinically relevant multidrug resistant strain provides a valuable framework for exploring NagA as a potential target in antibiotic development [5, 13, 15, 16]. Given its essential role in cell wall biosynthesis, kpNagA could serve as a strategic point of intervention in combating infections caused by resistant *K. pneumoniae*.

## Accession Numbers

Atomic coordinates and structure factors for the reported crystal structures have been deposited with the Protein Data Bank under accession codes 9UP6.

## Supplemental Materials

Supplementary data for this paper are available on-line only at http://jmb.or.kr.



## Figures and Tables

**Table 1:** Data collection and refinement statistics.

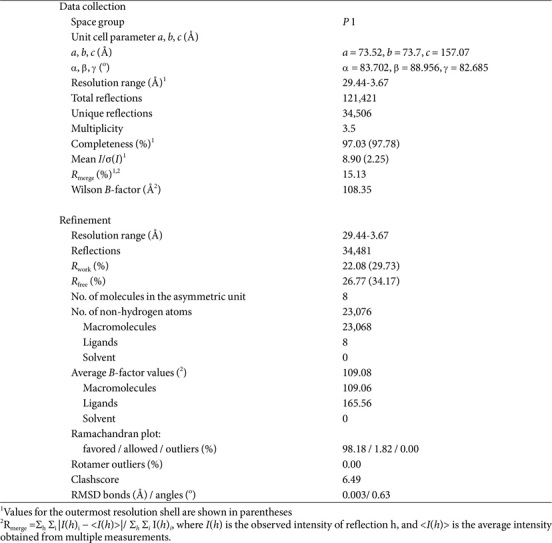

**Table 2:** Summary table of dimer interface properties assessed by PDBePISA.

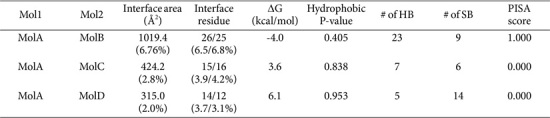

**Table 3:** Structural similarity search using DALI [31].

Proteins (accession numbers)	Z-score	RMSD (Å)	Identity (%)	References
NagA from *P. Multocida* (6JKU)	62.0	0.9	53	[14]
NagA from *E. coli* (2P50)	59.2	0.6	86	[7]
NagA from *V. cholerae* (3EGJ)	61.6	1.0	62	Unpublished
NagA from *B. subtilis* (2VHL)	53.9	1.7	29	[9]
NagA from *T. maritima* (1O12)	50.7	1.7	27	Unpublished
AMDHD2 from *Homo sapiens* (7NUT)	50.6	2.1	33	[10]
NagA from *M. smegmatis* (6FV3)	45.7	1.7	34	[13]
