# Supplementary material for: Structural Basis for Tetramerization of Klebsiella pneumoniae N-Acetylglucosamine-6-Phosphate Deacetylase
Source: J Microbiol Biotechnol. 2025 Aug 26;35:e2505019. doi: 10.4014/jmb.2505.05019 (PMC12409433; doi:10.4014/jmb.2505.05019)
Supplement: Supplementary file 1 [file jmb-35-e2505019-supple.pdf]

J. Microbiol. Biotechnol. 2025. 35: e2505019

https://doi.org/10.4014/jmb.2505.05019

Structural Basis for Tetramerization of *Klebsiella pneumoniae N*-Acetylglucosamine6-Phosphate Deacetylase

So Yeon Lee1,2 and Hyun Ho Park1,2*

[1]College of Pharmacy, Chung-Ang University, Seoul 06974, Republic of Korea

[2]Department of Global Innovative Drugs, Graduate School of Chung-Ang University, Seoul 06974, Republic of Korea

* To whom correspondence should be addressed.
Email: xrayleox@cau.ac.kr

Supplementary Figures

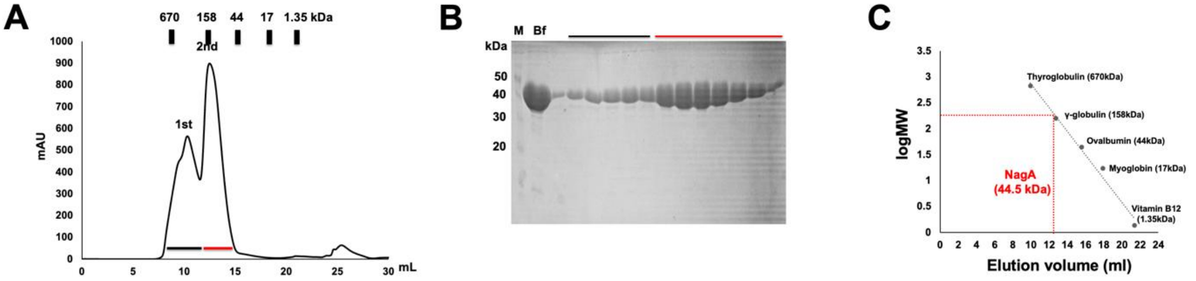

Fig. S1. (A) Size-exclusion chromatography (SEC) profile of purified kpNagA, showing two major peaks. The second peak, corresponding to the predominant species, is indicated in red. **(B)** SDS-PAGE analysis of SEC fractions from both peaks. The consistent band at ~42 kDa confirms purity and expected monomeric size. **(C)** SEC calibration curve plotted as log molecular weight versus elution volume. The elution volume of kpNagA is marked in red, corresponding to ana apparent tetrameric assembly.

Supplementary figure S2.

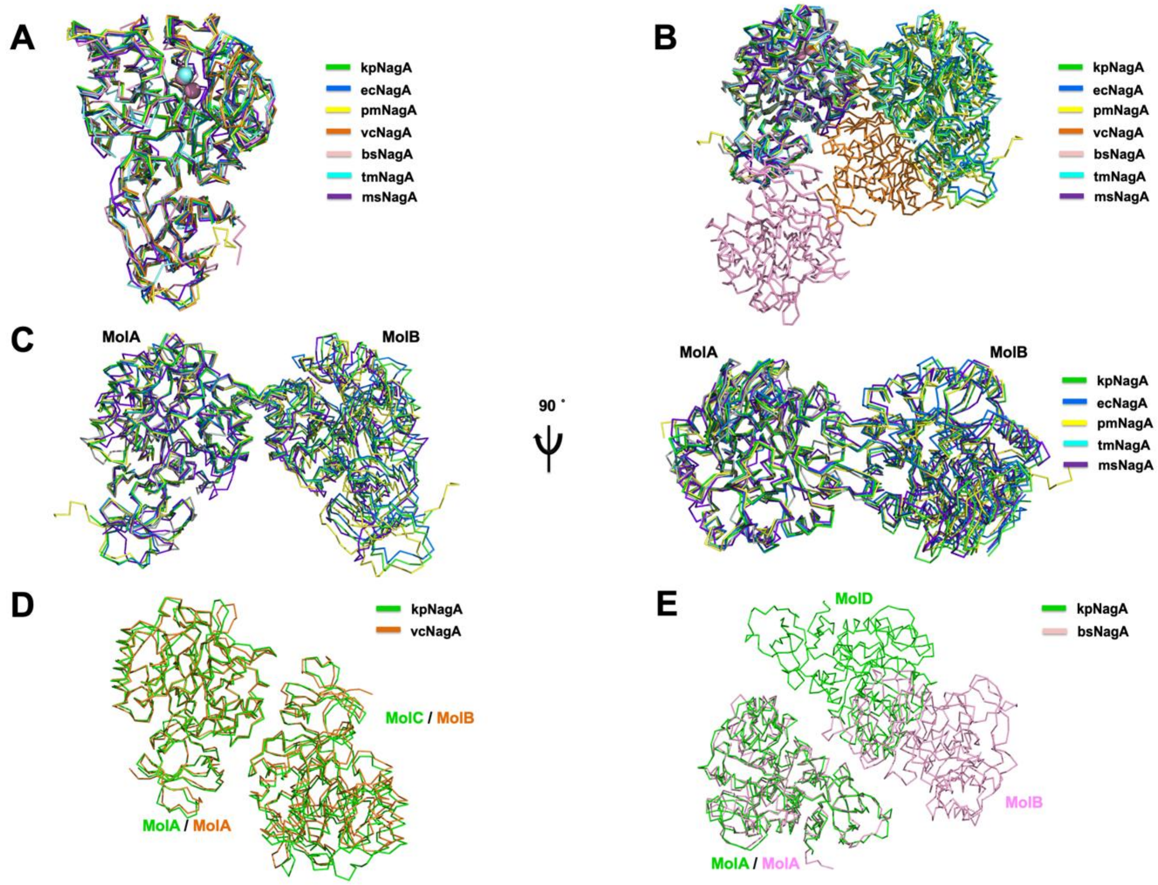

Fig. S2. (A) Superimposed monomeric structures of NagA homologs from various species, illustrating conserved overall fold. **(B)** Superimposition of dimeric NagA homologs, highlighting structural similarity among canonical dimers. **(C)** Structural overlay of MolA-MolB dimer from kpNagA, ecNagA, pmNagA, tmNagA, and msNagA. **(D)** Structural alignment of vcNagA dimer with the MolA-MolC interface of kpNagA. **(E)** Structural alignment of bsNagA dimer with the MolA-MolD dimer of kpNagA.
